# Earliest directly dated rock art from Patagonia reveals socioecological resilience to mid-Holocene climate

**DOI:** 10.1126/sciadv.adk4415

**Published:** 2024-02-14

**Authors:** Guadalupe Romero Villanueva, Marcela Sepúlveda, José Cárcamo-Vega, Alexander Cherkinsky, María Eugenia de Porras, Ramiro Barberena

**Affiliations:** ^1^ Consejo Nacional de Investigaciones Científicas y Técnicas (CONICET), Instituto Nacional de Antropología y Pensamiento Latinoamericano, Ciudad Autónoma de Buenos Aires, Buenos Aires, Argentina.; ^2^ Department of Social Sciences, Universidad de Tarapacá, Iquique, Chile.; ^3^ Laboratorio de Espectroscopía Vibracional, Universidad de Chile, Santiago de Chile, Chile.; ^4^ Center for Applied Isotope Studies, University of Georgia, Athens, GA, USA.; ^5^ Instituto Argentino de Nivología, Glaciología y Ciencias Ambientales–CCT Mendoza CONICET, Mendoza, Argentina.; ^6^ Centro de Investigación, Innovación y Creación (CIIC-UCT), Facultad de Ciencias Sociales y Humanidades, Universidad Católica de Temuco, Temuco, Chile.; ^7^ Instituto Interdisciplinario de Ciencias Básicas (ICB), Consejo Nacional de Investigaciones Científicas y Técnicas (CONICET), Universidad Nacional de Cuyo, Mendoza, Argentina.

## Abstract

The timing for the evolution of the capacity to inscribe the landscape with rock art has global relevance. While this was an in-built capacity when *Homo sapiens* first colonized the Americas, the heterogeneous distribution of rock art shows that it was a facultative behavior arising under unknown socioecological conditions. Patagonia was the last region to be explored by humans. While its rock art is globally important, it remains largely undated by absolute methods. We report the earliest set of directly radiocarbon-dated rock art motifs from the archaeological site Cueva Huenul 1 (northwestern Patagonia, Argentina), starting at 8.2 thousand years before the present (ka B.P.), predating previous records by several millennia, and encompassing over 3 ka (~130 human generations). This mid-Holocene “rock art emergence” phase overlaps with extremely arid conditions and a human demographic stasis. We suggest that this diachronic rock art emerged as part of a resilient response to ecological stress by highly mobile and low-density populations.

## Teaser

Rock art in northern Patagonia emerged during the arid mid-Holocene as a resilient response to ecological stress.

## INTRODUCTION

The debate on the origins of rock art across continents is linked to the evolutionary development of cognitive abilities, the emergence of symbolic behaviors, and the ensuing sociodemographic trajectories (*1*–*4*). Worldwide, archaeology seeks to establish the timing and spatial patterning of landscape inscription with images as a tool to understand its social, adaptive, and evolutionary roles (*5*–*7*). The simultaneous material and symbolic quality of these visual expressions makes them a unique indicator of landscape learning, social dynamics, information flow, and construction of territories by mobile groups at multiple scales. While recent interdisciplinary approaches make the robust dating of rock art increasingly feasible (*6*–*10*), the absolute temporal placing of motifs still hampers our understanding of the varying regional origins and roles of rock art.

In South America, chronological analysis of rock art has largely relied on relative sequences based on iconographic analysis with few absolute dates on painted or engraved images (*9*, *11*–*13*). Patagonia constitutes the southernmost tip of the Americas and was the last continental region to be settled by dispersing modern humans during the Late Pleistocene (*14*–*16*). Its unique biogeographic context coupled to an excellent preservation of paleoecological and archaeological evidence makes it a primary region to understand the peopling of the Americas and the role of symbolic marking of the landscape by means of rock art (*15*, *17*). With few exceptions (*8*, *13*, *18*, *19*), nonetheless, Patagonian rock art remains only dated by relative methods or by absolute dates on contextually associated remains (*11*–*13*, *18*–*25*).

**Fig. 1.**
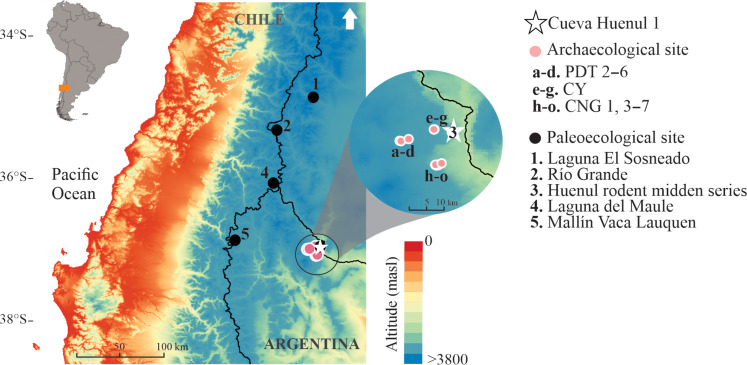
Location of CH1, other sites with rock art in northern Neuquén Province (Argentina), and paleoecological sites from northwestern Patagonia. Digital credits: M.E.d.P.

As part of a regional project combining the study of paleoecological history, systematic landscape survey, and intensive dating of human occupations in stratified contexts (*26*–*29*), we present the earliest directly dated rock art from northern Patagonia (Neuquén Province, Argentina; Fig. 1). Four diagnostic motifs from the archaeological site Cueva Huenul 1 (CH1) were archaeometrically characterized and securely dated by Accelerator Mass Spectrometry (AMS). Building on this case, we develop a multiscalar approach connecting successive analytical levels: At a local scale, we present a formal, compositional, contextual, and chronological study of diachronic human practices of place-marking and transmission of information; at a macroregional scale, we situate the evidence of early rock art production in northern Patagonia in the context of paleoecological trends since the Late Pleistocene, combined with a radiocarbon-based approach to demographic trends in the South American drylands (*17*, *30*, *31*). By presenting solid site-scale evidence on intergenerational transmission of knowledge situated in a macroregional socioecological framework, our case study contributes to ongoing global discussions on the origins and social and adaptive roles of rock art.

## RESULTS

### Picturing a place: CH1 archaeological site

**Fig. 2.**
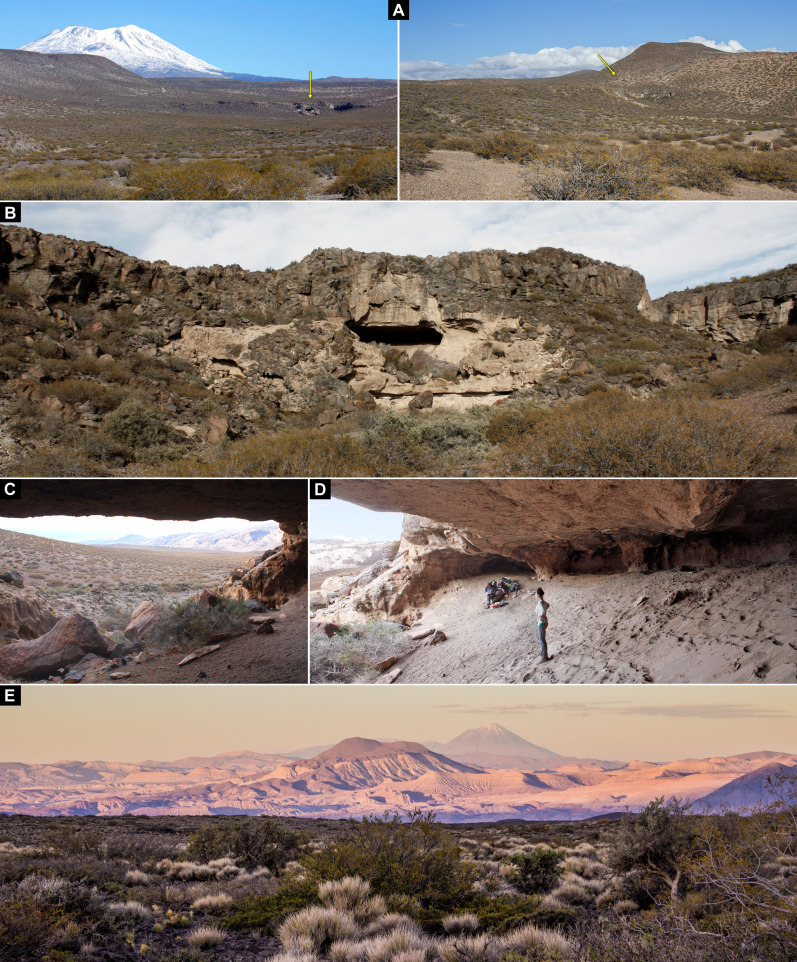
CH1 environment and landscape. (**A**) Emplacement of CH1 (yellow arrow) in a volcanic landscape within the Monte Desert. (**B** to **D**) Views of the cave’s geology and topography. (**E**) View from CH1 of the volcanic landscape of northwestern Patagonia. Photo credits: G.R.V.

CH1 is located at 1000 m above sea level in the inland deserts of northwestern Patagonia in Argentina, South America (Figs. 1 and 2). This region lies at 36°S eastward of the Andes within the South American Arid Diagonal (SAAD), a major climatic and biogeographic region encompassing most of the drylands in South America (*32*). The Andes is a topographic barrier to the prevailing westerly storm tracks, affecting the patterns of atmospheric circulation and imposing a steep west-east gradient on precipitation and effective moisture (Fig. 1) (*33*). At 36°S, annual precipitation varies from approximately 1100 mm close to the current Argentina-Chile border to 200 to 150 mm in the core of the SAAD. Most of the precipitation (~75%) falls during winter and is brought from the Pacific Ocean by the Southern Westerlies, while summer precipitation related to Atlantic moisture is negligible in this area. Vegetation distribution follows this gradient, from forest communities west of the Andes (Chile) and the Andean slopes in Argentina to the grass steppes of the eastern Patagonia Province, characterized by a low shrubby steppe intermingled with tussock grasses (*27*). As altitude and precipitation decrease toward the eastern lowlands, the ecotone with the shrub-steppes of the Monte Province that characterize drylands at this latitude develops (Fig. 2).

**Fig. 3.**
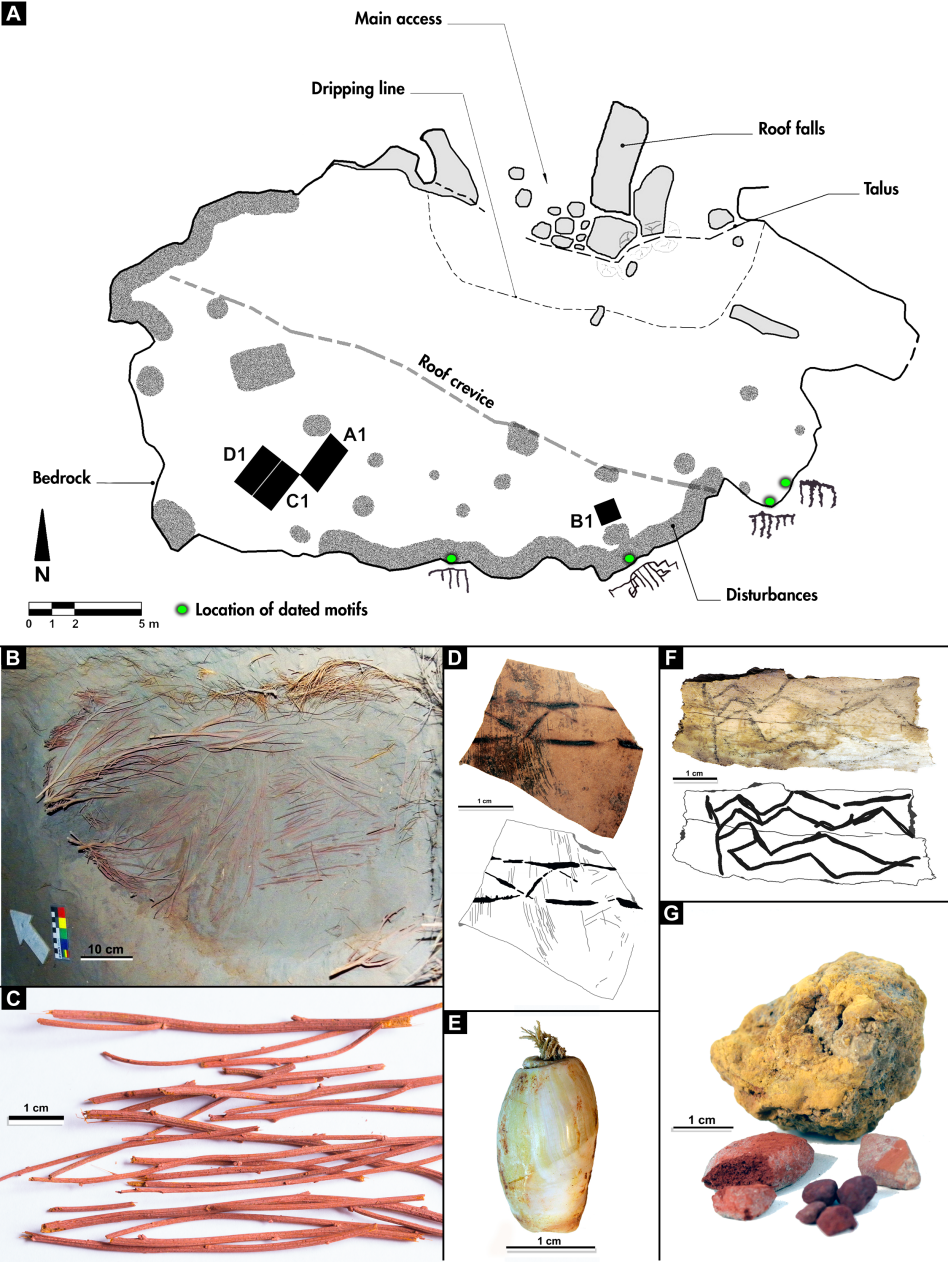
CH1 site plan and special findings. (**A**) Excavation units at CH1. (**B** and **C**) General and detailed view of pit structure filled with vegetal remains of *S. aphylla* stained with red ocher. (**D**) Pyro-engraved gourd. (**E**) Perforated shell bead. (**F**) Decorated guanaco (*Lama guanicoe*) bone. (**G**) Pigments. Each image has an individual metric scale. Photo and digital credits: G.R.V. and R.B.

CH1 covers a large habitation area of 630 m^2^ that is unparalleled at the macroregional level (Figs. 2, B to D, and 3A) (*26*). The cavity is formed by erosion at the contact between the ignimbrites of the Tilhué Formation at the bottom, where the paintings were made, and basalts of El Puente Formation forming the roof of the cave (Fig. 2B) (*34*). The excavations of CH1 resulted in the recovery of over 5500 lithic artifacts and 8800 bone specimens, principally flaked stone artifacts and guanaco bone assemblages with virtually no ceramics. CH1 has a long and well-dated chronostratigraphic sequence spanning 12 thousand years (ka) showing discrete and discontinuous phases of site formation. A stable microenvironment with predominantly dry conditions within the cave has produced excellent preservation of organic materials including megafauna dung and macrobotanical remains (*34*). Moreover, CH1 provides a remarkable set of unusual portable art artifacts channeling visual communication (Fig. 3, B to G), such as perforated shell beads, decorated guanaco bones, and pyro-engraved gourds (*35*). Other findings emphasize color display such as an unusual pit structure filled with vegetal remains of a desert shrub (*Senna aphylla*) densely stained with red ocher (*35*). In addition, large amounts of pigments of various colors, sizes, and preparation stages recovered in stratigraphy throughout the occupational sequence potentially indicate the in situ decoration of many of these items, as well as the preparation of pigments used for rock art production.

CH1 has a wide view of the surrounding landscape (Fig. 2), although it does not have important intervisibility with other archaeological sites with redundant occupations and/or rock art (Fig. 1). Most of these sites are caves and rock shelters with considerably smaller habitation areas than CH1 and presenting residential occupations largely bracketed to the last 2 ka (*29*). Some of these locales, such as El Ciénego and Paso de las Tropas, were marked with rock art, although less redundantly and with lesser formal and technical variety than recorded for CH1 (*28*). The site Cueva Yagui, on the other hand, also presents a long and discontinuous occupational sequence starting at 8.5 ka, albeit characterized by a higher occupational intensity than CH1 as indicated by lithic artifacts and tools discard rates, abundance of ceramics, and intensity in the processing of faunal preys, particularly during the last 2 ka (*36*). Human use of CH1, Cueva Yagui, and the other sites in the region was linked through circuits of mobility and information networks, as suggested by the surface evidence, stratigraphic contexts (*29*, *34*, *37*), and the similarities in the rock art repertoires (*28*). However, their diverse occupational histories suggest that these places—many of them with rock art—occupied different positions within past human networks of mobility and settlement (*35*, *36*).

### Placing pictures: The rock art repertoire at CH1

The central portion of the cave’s internal wall and part of the ceiling is covered with 895 discrete painting events operatively grouped in 446 motifs distributed across the site (fig. S1) (*35*). CH1 is the most notable place for pigment-based rock art production in northwestern Patagonia and neighbor areas of central-western Argentina and central Chile (*22*, *38*, *39*). The painted repertoire is formally exceptional with images depicting an outstanding variety of shapes, colors, and sizes, many of which are seldom found together (fig. S1). The assemblage is dominated by nonfigurative designs painted in different hues of red, chemically characterized as hematite. Most of them depict elemental geometric shapes (strokes, dots, circles, and lines), while others portray more complex and larger designs (parallel lines, reticulates, polygons, and cruciforms). Albeit less frequent, figurative designs such as anthropomorphs (human silhouettes and a face) and zoomorphs (silhouettes of guanaco and choique—*Rhea pennata*) are also present, as well as motifs painted in different hues of white, yellow, and black, either used individually or in bichrome compositions (fig. S1). Representations portraying dynamic group activities are another rare feature of CH1’s repertoire in the regional and macroregional context.

The large number of superimpositions recorded, the presence of three different weathering degrees, and the formal variability observed support the interpretation of diachronic rock art painting phases (fig. S1). A thorough chronological contextualization by relative methods suggests that most of the motifs were painted during the Late Holocene (*35*), based on the formal similarities with late Patagonian rock art styles such as *Grecas* (Fret) and *Paralelas* (Parallels) (*20*, *40*–*43*). However, given the ancient and discontinuous character of the stratigraphic sequence containing ocher remains, earlier rock art production in CH1 remained a distinct possibility.

### Anchoring images in time: The dates for rock art in CH1

**Fig. 4.**
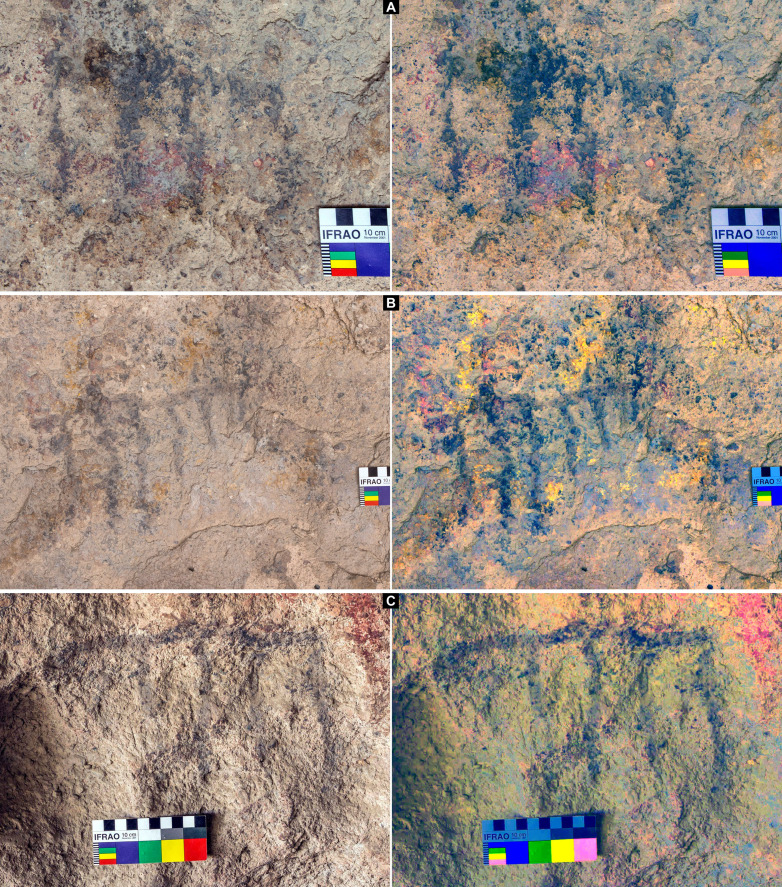
Dated rock art paintings from CH1. (**A**) Motif UT3-M37. (**B**) Motif UT3-M48. (**C**) Motif UT5-S4-M7. Original photograph and digital enhancement with Dstretch_ybk. Photo credits: G.R.V.

**Fig. 5.**
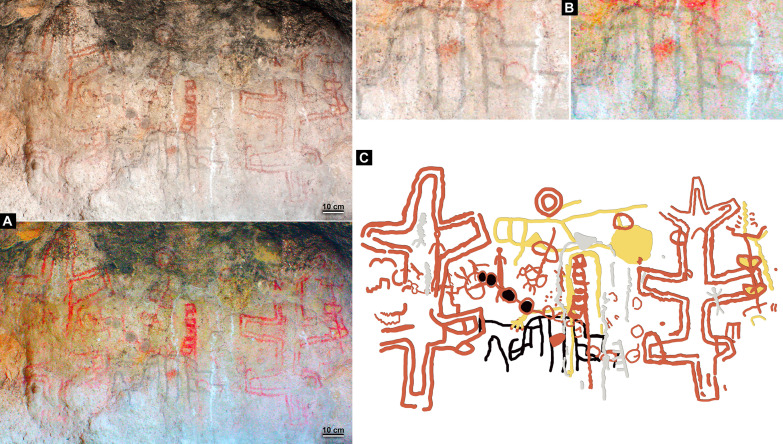
Dated rock art motif UT5-S2-M19 from CH1. (**A**) Original photograph and digital enhancement with Dstretch of the complete rock art panel. (**B**) Original photograph and digital enhancement with Dstretch of the dated black comb-shaped motif. (**C**) Digital tracing of the complete rock art panel showing the dated black comb-shaped motif underlaying a series of superimpositions. Photo and digital tracing credits: G.R.V.

The four dated motifs are nonfigurative and were operatively classified by the same observer (G.R.V.) as comb-shaped, because their basic morphology shows several parallel vertical lines extending from a perpendicular horizontal line, such as spikes extended from the strip of a comb. However, slight differences are observed between them [design types A (simple) versus B (complex)] (Figs. 4 and 5). The four motifs are registered as reddish black in color (10R-2.5/1 Munsell Soil Chart), all showing the highest degree of weathering defined for the site’s paintings (*35*). Only one motif (UT5-S2-M19) is implied in a complex series of superimpositions involving motifs of different morphology, color, and size (Fig. 5).

**Fig. 6.**
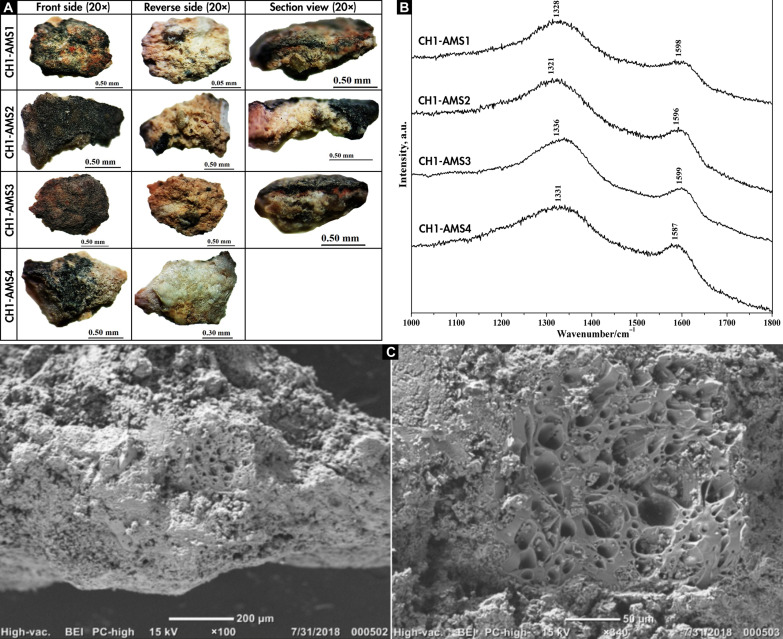
Optical microscopy, Raman, and SEM-EDX analysis for CH1. (**A**) Optical microscopy and (**B**) Raman for the dated motifs of CH1. (**C**) SEM-EDX images of black paint sample CH1-AMS2. In (C), the left image (×100) shows the presence of plant cells at the center included in other mineral concretions. The right image (×340) shows plant cells with more detail. Photo and digital credits: G.R.V., M.S., and J.C.-V.

A multi-analytical archaeometrical approach allowed assessing the paint composition to secure ulterior AMS dating (Table 1). By means of optical microscopy (Fig. 6A and fig. S2), we ruled out the presence of organic matter either on, within, or below the paint layer, which may contaminate the samples. We provide the microstratigraphy of one sample that shows the presence of three layers: the bedrock support, a layer of superimposed paint, and a thin layer composed of translucent particles (fig. S2). The black paint layer is of relatively large thickness between 30 and 50 μm with particles of intense color, shiny, and dull (Fig. 6). The Raman analysis of four samples of black paints shows broad vibrational signals of medium high intensity, between 1590 and 1335 cm^−1^ (Fig. 6B), the bands respectively assigned to the vibrational modes of the G and D bands of amorphous carbon (*44*, *45*). The absence of bands around 960 cm^−1^ (Fig. 6B), typical of the phosphates associated to bone composition, suggest that the identified carbon does not come from bone (*44*, *45*).

**Table 1.** Archaeometric and chronometric results for the dated motifs of CH1.

Sample ID	CH1-AMS2	CH1-AMS4	CH1-AMS1	CH1-AMS3
Motif ID	UT3-M48	UT5-S4-M7	UT3-M37	UT5-S2-M19
	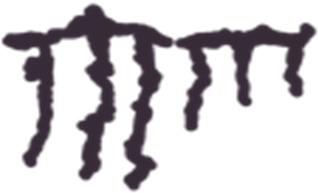	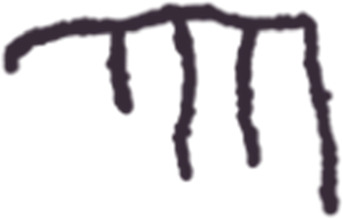	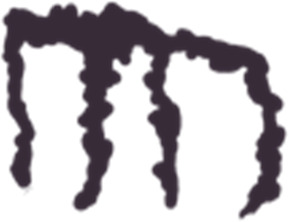	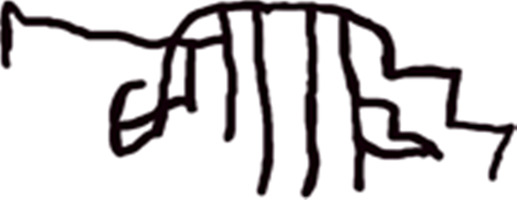
Motif type and design	Comb-shaped			
	Design A	Design A	Design A	Design B
Weathering degree	Highest (n° 3)			
Color and hue (Munsell)	Black (10R-2.5/1 reddish black)			
Archaeometric characterization (optical microscopy, Raman, SEM-EDX)	Absence of inclusions either on, within, or below the paint layer			
	Amorphous carbon (possible vegetal origin)			
AMS Laboratory Code	UGAMS #29468	UGAMS #29470	UGAMS #29467	UGAMS #29469
Mass of carbon sample combusted (μg C)	58	46	11.5	4
δ^13^C (‰) relative to PDB	−18.0	−23.47	−24.3	−24.51
Trophic position of the material dated	C_3_-CAM plant mix?	C_3_ plant	C_3_ plant	C_3_ plant
^14^C yr B.P. (±1σ)	6830 ± 50	5360 ± 50	4730 ± 110	3010 ± 160
Percentage of modern carbon (pCM)	42.73 ± 0.28	51.31 ± 0.33	55.51 ± 0.73	68.75 ± 1.37
Calibrated age B.P. (±2σ, 95.4%)	7565–7728	6239–6271	5629–5643	2761–3477

**Fig. 7.**
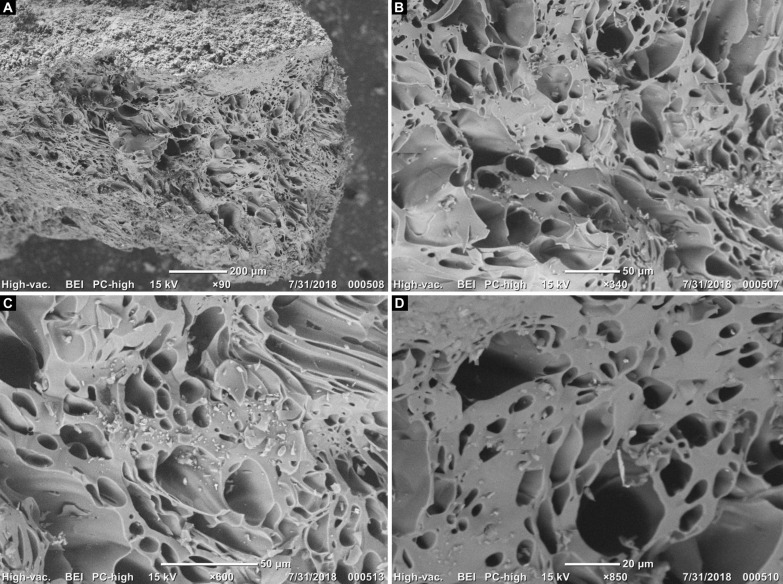
Presence of plant cells (carbon) in the CH1-AMS4 sample of black paint from CH1. Magnification: ×90 (**A**), ×340 (**B**), ×600 (**C**), and ×850 (**D**). Photo and digital credits: G.R.V. and M.S.

Scanning Electron Microscopy (SEM) with Energy Dispersive X-Ray Analysis (EDX) showed a vegetal origin of the carbon with the observation of plant cells in the four dated samples [Figs. 6C and 7 and figs. S3 (A to C), S4, and S5]. The spectra (fig. S3, A to C), the images (Fig. 7C and fig. S4), and the elemental cartography (fig. S5) confirm the presence of carbonaceous material inserted in an aluminosilicate matrix, as part of a possible intentional mixture, surrounded by a heterogeneous layer composed of sulfur and calcium—probably a salt. While precise identification of the wood was impossible due to small size and lack of comparable references (*46*), it is likely that shrubby species such as *Prosopis* spp., *Larrea* sp., or *Schinus* sp. were used, because they would be the main available sources of wood in the Monte desert as shown by the early and mid-Holocene plant macrofossil record from rodent middens from CH1 (*27*).

Together, images and chemical analyses demonstrate that the black pigment is amorphous carbon resulting from the incomplete combustion of organic compounds (Figs. 6 and 7), indicating the possibility to directly date the amorphous carbon of the rock art paintings with ^14^C AMS. Sampling involved taking complete wall fragments with the presence of paint, which were then processed in the laboratory with specific protocols to extract the organic material necessary for dating.

Detailed results of the archaeometric characterization and radiocarbon dating of the four black painted motifs from CH1 are summarized in Table 1. The association between the samples dated (amorphous carbon) and the events of interest (painting of the rock surfaces) is a central methodological concern (*6*, *47*, *48*). The samples CH1-AMS1, CH1-AMS3, and CH1-AMS4 display δ^13^C values between −23 and −24‰, consistent with those reported for C_3_ plants globally and in Patagonia (*49*, *50*). Considering that there are no long-lived trees in these deserts where available woody taxa are basically shrubs such as *Larrea*, *Prosopis*, or *Schinus* (*27*), the Old Wood factor would not be important (*51*). Sample CH1-AMS2 has a more enriched value of −18.0‰, lying within the range of variation that we have measured for a large sample of guanaco bone collagen from the site CH1 itself (*52*). However, as mentioned above, the absence of bands typical of the phosphates (around 960 cm^−1^) suggests that the carbon does not come from bone. The alternative that we consider most plausible is a combination of a C_3_ shrub—from which the wood observed comes from—with a cactus [Crassulacean acid metabolism (CAM) photosynthetic pathway] such as *Maihueniopsis darwini*, with local values between −13 and −10‰ (*53*). This cactus has been abundantly recorded in CH1’s macro-botanical assemblage since the early Holocene (*37*, *54*), including fragments stained with red ocher (fig. S6). Cacti slime was used as a binder in northern Argentina (*55*, *56*), as well as ethnographically in the Americas (*57*). While we cannot establish with precision the combination of all the materials used, particularly for sample CH1-AMS2, we consider this to be the most parsimonious explanation to account for the Raman and isotope results.

The mass of carbon sample combusted (μg C) allows suggesting that the samples CH1-AMS1 (11.5 μg), CH1-AMS2 (58 μg), and CH1-AMS4 (46 μg) are reliable (Table 1). Sample CH1-AMS3, on the other hand, presents very low mass of carbon combusted (4 μg) and is considered unreliable given the potential for contamination with younger carbon. Because we question the potential source(s) of carbon in this sample, we do not consider that it accurately dates a painting event and therefore exclude from following analyses (*47*, *58*). The earliest dated image from CH1 [CH1-AMS2: 6830 ± 50 radiocarbon years before the present (^14^C yr B.P.), unmodeled 2σ = 7779 to 7578 calibrated years before the present (cal yr B.P.), median = 7660 cal yr B.P.] is the oldest directly radiocarbon-dated pigment-based rock art record in South America, predating by several millennia previously published information (*8*, *9*, *11*–*13*). In all these cases, the ages date the death of the plants burnt and/or used as a binder to prepare the pigments, thus providing a terminus post quem—maximum ages—for the execution of the motifs.

### Rock art production phase and occupational history of CH1: A Bayesian model

As part of a program of systematic dating of the deposits of CH1 (*26*, *59*), we obtained 16 radiocarbon dates for the stratigraphic sequence of the site, in addition to the four dates for rock art (table S1). We develop a Bayesian model in OxCal (*60*) of the site’s occupational history that defines four sequential occupation phases based on previous chronostratigraphic and contextual evidence (dates in table S1 and OxCal code in text S1). The resulting model is robust, with an overall agreement index of 98, thus providing reliable estimations of the boundaries and intervals for each phase (fig. S7).

**Fig. 8.**
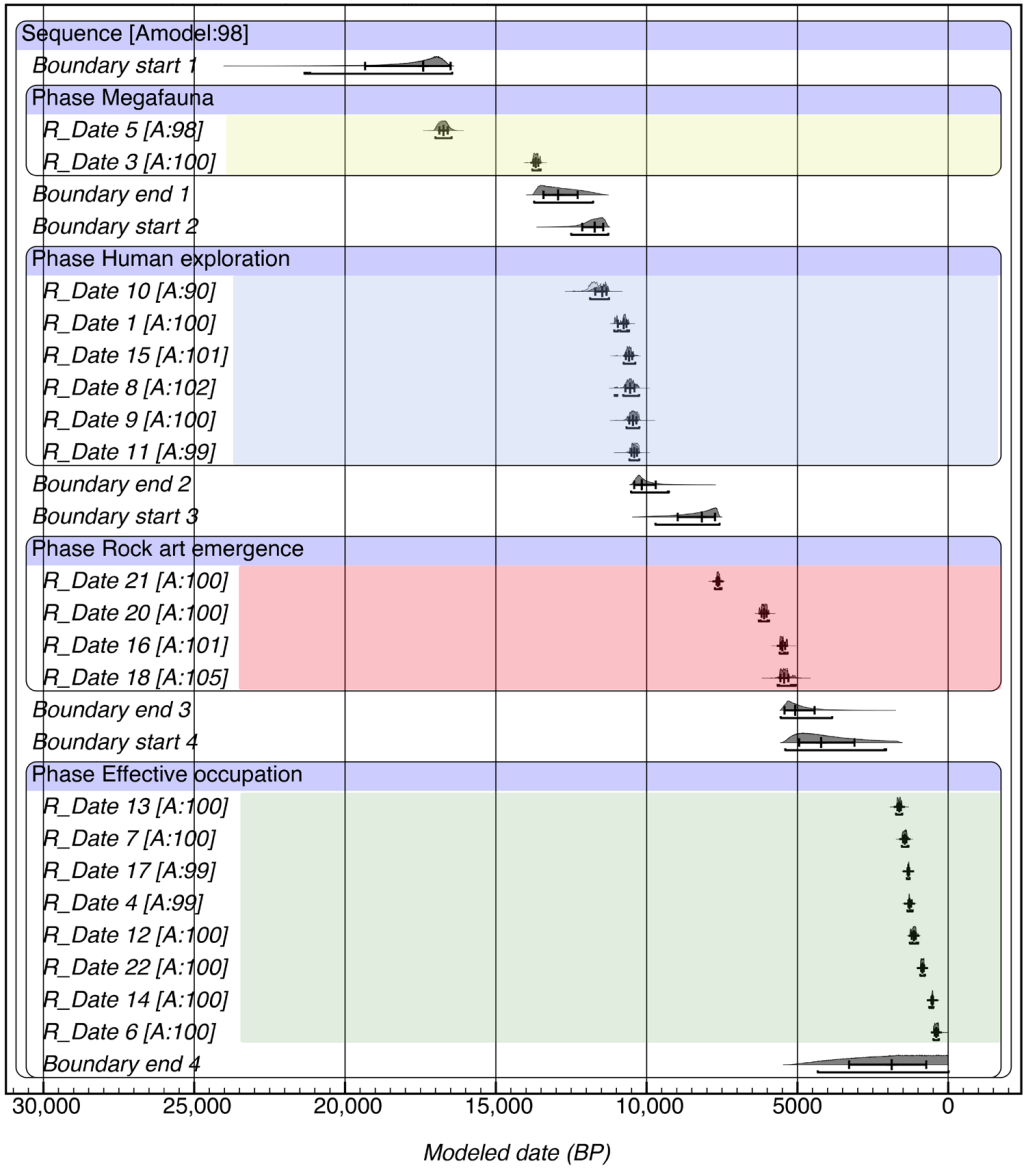
OxCal Bayesian model for the sequence of occupational phases in CH1. Bars under probability distributions indicate 95.4% probability range. Digital credits: R.B. Produced with OxCal v4.4.4 (*111*) with atmospheric data from (*105*).

The sequence of CH1 discontinuously spans ~18 ka between the Late Pleistocene and recent times (Fig. 8). The first “megafauna phase” is defined by two dates bracketing a dung-rich deposit at the base of the stratigraphic sequence with a median interval of 4683 years and start and end boundaries at 17,407 to 12,934 cal yr B.P. This phase is defined by the presence of extinct sloths at the cave during the terminal Pleistocene glaciations and does not show human occupations. The second phase of “Human exploration” is built on six dates from different anthropogenic materials (guanaco bones with cut marks, charcoals from hearths, and grasses from a bedding structure) and represents the first human presence at the site and the region. The median interval of this phase is 1620 years, with medians for start and end located at 11,721 and 10,162 years B.P. (Fig. 8).

Then, there is a chronostratigraphic hiatus followed by the third phase of “rock art emergence” defined by the three secure dates for painted motifs plus one additional overlapping date on ocher-covered plants (*S. aphylla*) filling a pit structure (Fig. 8), which is the only stratigraphic evidence pertaining to the mid-Holocene at CH1 (*34*). The rock art emergence phase has a modeled start at 8171 years B.P. and an end at 5074 years B.P. with a median interval of 3246 years. Considering a conventional estimation of 25-year human generation (*17*, *61*), this interval would represent 130 generations. Considering the strong visual, technical, material, and locational affinity between the three dated motifs, the Bayesian modeling of the dates for rock art suggests a sustained behavior of intergenerational transmission of information tied to a topographically unique landscape feature such as CH1. Last, eight dates on different cultural materials recovered from the excavations allow building an “effective human occupation” phase (*62*), showing more intense local and regional occupations spanning over 1.5 ka during the Late Holocene.

### Images in context: Paleoenvironmental and sociodemographic scenarios

**Fig. 9.**
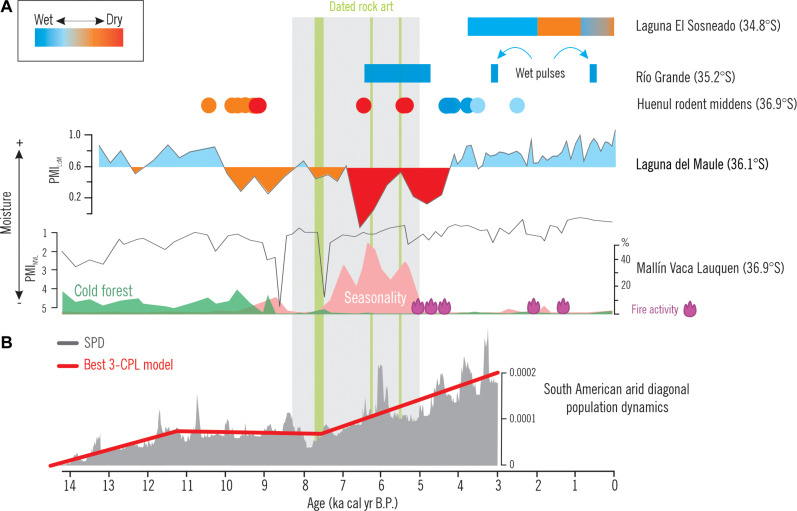
Temporal trends for human occupations and rock art production in CH1 within demographic and paleoenvironmental synthesis (34°S to 36°S) from northwestern Patagonia since the Late Pleistocene. (**A**) The figure includes the glacial advances in Río Valenzuela (*75*, *76*), pollen record of Laguna El Sosneado (*110*), Huenul rodent midden series (*27*), Pollen Moisture Index of Laguna del Maule (*63*), and Pollen Moisture Index and charcoal record of MVL (table S2). The vertical green bands represent the calibrated age range of the rock art dates, while the gray vertical band encompasses the full rock art emergence phase. (**B**) Summed probability distribution of the ^14^C dates from archaeological sites in the SAAD (gray silhouette) and best three continuous piecewise linear (CPL) fitted model (red line) (*17*). Digital credits: M.E.d.P.

Seeking to explain long-term behavioral phenomena, we now situate the chronological record from CH1 in a broad paleoecological and sociodemographic framework for northwestern Patagonia. A synthesis of the paleoecological trends shows that a west-east moisture antiphase characterized the climatic and environmental conditions during the Late Pleistocene–Early Holocene (until 10 ka B.P.) with generalized colder but wetter/drier conditions along the western/eastern side of the Andes (Fig. 9) (*63*, *64*). In the eastern lowlands, however, the record of the Huenul rodent midden series (HU) (*27*) indicates prevailing more arid than present conditions between 10.4 and 9.4 ka B.P.—in agreement with most subtropical records along the SAAD (*65*–*67*). Slightly drier than present conditions prevailed in the highlands of northwestern Patagonia during the early Holocene—10 ka B.P. onward—(*64*) and turned into extremely dry in the Andes (*63*) during the mid-Holocene, as well as across the entire SAAD.

The mid-Holocene was indeed the driest period of the Holocene for many regions within the SAAD, making its impact on ancient human societies a central topic in South American archaeology (*27*, *65*, *67*–*69*). Multiple paleoecological proxies indicate that enhanced aridity in these deserts would have increased landscape fragmentation, expressed as large tracts of the landscape acting as temporary or continuous barriers for humans (*70*). The barrier-character would imply infrequent human use associated to elevated risks involved in traversing or occupying very dry and unpredictable areas (*71*). While it remains as a distinct possibility that the most arid tracts of the landscape were either abandoned or occupied only as “passing-through places” (*70*), the macroregional record suggests that human populations occupying the central-western part of the SAAD coped with mid-Holocene aridity from a demographic perspective. This may have been achieved by relocating in space (*65*), changing settlement and subsistence patterns (*72*, *73*), extending social networks (*39*, *71*), producing technological innovations, or combinations thereof.

Along the eastern side of the Andes, wetter and warmer conditions than before were established between 8.6 and 5.3 ka B.P. associated to a high precipitation variability with extremely dry phases around 8.6 and 7.5 ka and a strongly seasonal climate regime with rainy winters and extremely drier than present summers, particularly between 7.3 and 5 ka B.P. (Fig. 9) (*64*). The episodic evidence of the lowland HU record also suggests extremely dry local conditions in the Monte desert (*27*). The absence or scarcity of rodent middens in space/time blocks has been associated with a lack of resources required for rodent survival in each environment/time (e.g., Atacama Desert/mid-Holocene) (*74*), thus supporting the prevalence of extremely dry mid-Holocene conditions around Cueva Huenul. At 35°S, the occurrence of Neoglacial advances at the Río Valenzuela basin (*75*, *76*) suggests the establishment of wet conditions between 6.5 and 4.8 ka B.P., probably associated to the high precipitation variability recorded further south. However, the discontinuous nature of the geochronological evidence at this basin may reflect subcentennial wet pulses superimposed to the millennial-to-centennial widespread arid conditions prevailing across the SAAD. This paleoecological framework shows prevailing regional dry conditions during the initiation and development of the rock art emergence phase in CH1 (Fig. 9).

We use a comprehensive database of radiocarbon dates for human occupations in the SAAD (*17*) as a proxy of the long-term macroregional population dynamics (*77*–*79*). The distribution of dates suggests that after a period of rapid population growth (4.15% per generation) between 14 and 10.8 ka B.P., likely due to the successful exploration and colonization of diverse and uncontested niches by early human societies resulting in spread dynamics, the period between 10.8 and 7 ka B.P. saw a contrasting trend with a large-scale stasis in the radiocarbon record and slight estimated population decrease of −0.05% per generation (Fig. 9) (*17*). Because the rock art emergence phase has a modeled start at 8.2 ka B.P., its first part overlaps with the demographic stasis recorded for South American deserts. This is consistent with larger trends for South America, showing a relatively constant population size between 9 and 5.5 ka, indicating that mid-Holocene population density was in the lower range of estimates for the density of worldwide hunter-gatherer populations (*80*). Moreover, it has been suggested that demographic trends for South America fall below expectations for population growth between 8.6 and 6 ka B.P., indicating that a periodic and substantial population deflation is apparent in the archaeological ^14^C record on a continental level (*81*).

Thus, available information allows situating our chronometric results for the rock art emergence phase in northwestern Patagonia on a macroregional socioecological scenario. Paleoecological records suggest a period of enhanced aridity at millennial-to-centennial scale with the potential occurrence of wetter pulses at the subcentennial scale that would have ameliorated—but not reversed—prevailing ecological conditions (*27*, *66*, *76*). Human demography, as inferred from the trends in a wide radiocarbon database, indicates a metapopulation that was failing to grow, probably due to the frequency of extreme climatic events (*81*). We suggest that a thinly distributed and highly mobile hunter-gatherer population in more unpredictable conditions than those prevailing during the previous exploration phase would have led to a vulnerable social landscape with a high cost of interaction across space (*71*).

## DISCUSSION

The discussion of when and why did humans start marking the Patagonian landscape with rock art was largely based on relative frameworks anchored by few reliable absolute dates, mostly falling in the Late Holocene (*8*, *12*, *18*–*20*, *23*, *24*). The sequentially dated comb-shaped motifs from CH1, grouped in a phase with a modeled start at 8.2 ka B.P. (table S1), provide robust empirical evidence on the origins of landscape marking by rock art in northwestern Patagonia. While these images could result from random mark-making and unintentional visual communication, the internal coherence of the diachronic assemblage of CH1 makes such an interpretation unlikely (*82*). However small the assemblage is, the set of diachronic motifs presents continuity in style, color, and materials used in its production. As modeled by Lewis and Laland (*83*), fidelity in cultural transmission leads to exponential growth in trait longevity and is key to build cumulative cultural repertories linking human groups across space and time (*84*, *85*). The expression of standardized images for over 3 ka implies a sustained intergenerational transmission of indigenous knowledge during the mid-Holocene, probably linked to the maintenance of collective memories (*86*, *87*).

Traditional knowledge tends to accumulate in previously occupied places that become “persistent places” (*88*) and attract subsequent occupations (*89*). Eventually, some of these places become cultural keystone places (*90*, *91*) established as the focus of activities such as collective hunting, social gatherings, and rituals that help to strengthen social networks (*71*, *92*). On the basis of our results, we suggest that the construction of CH1 as a cultural keystone place began during the Pleistocene-Holocene transition, characterized by a redundant occupational signal during the human exploration phase that spanned over 1400 years (11,652 to 10,204 cal years B.P.). During the mid-Holocene, this process was continued through sequential visits to the site, albeit on a different behavioral context. The focal activities recorded during this period relate to the marking of the cave walls with rock art motifs and by the deposition of a pit structure of unknown function filled with plants heavily stained with red ocher (Fig. 3, B and C). Unlike what is recorded for the Human exploration (Pleistocene-Holocene transition) and “Effective human occupation” (Late Holocene) phases, our excavations do not reveal deposition of materials related to daily human activities or residential occupations—faunal prey processing and lithic knapping—in CH1 during the rock art emergence phase (*26*, *34*, *37*). Hence, our multidisciplinary approach shows that the site was not abandoned—as many others in the region (*65*, *93*)—but experienced a behavioral shift during this time.

The emergence of rock art at CH1 is recorded since 8.2 ka B.P. against a challenging environmental background, where the ability to maintain connectivity and demographic viability would have been crucial (*71*, *94*–*96*). While reasons to visit CH1 during the mid-Holocene may have been manifold, we suggest that the standardized painting events—and other pigment-related activities—practiced over generations sought to maintain large-scale safety nets (*97*) by storing information rooted in collective memory and guaranteeing social preservation beyond oral tradition (*87*). The traditional knowledge associated with the motifs and the site continued to play an active role long after their production. We then propose that collective memory and social geography formed the contextual tapestry for rock art production at CH1, providing a place-based sense of identity.

Archaeological information suggests that other South American societies would have failed to respond adequately to mid-Holocene climate change, experiencing a widespread population decline due to landscape fragmentation produced by increasing aridity (*81*). While the iconographic continuity indicated by the motifs dated at CH1 may suggest that this was a successful strategy in Patagonia, we consider that this is not discernible yet, particularly during the initial part of the mid-Holocene (8 to 6.5 ka) that coincides with the earliest motifs. Previous studies based on radiocarbon databases and human mitochondrial DNA suggest a relatively continuous population growth in northwestern Patagonia starting at 7 to 5 ka (*30*, *31*), interpreted as a resilient human response to external impacts. However, as recently shown by demographic modeling by Gurven and Davison (*98*), periodic population crashes combined with plausible fertility reductions can reasonably generate averaged zero population growth as seen in the radiocarbon record from South American deserts (*17*, *26*, *80*, *81*). In this context, mirroring Gurven and Davison, we emphasize the role of intergenerational cooperation and cumulative culture in supporting the colonizing potential of human populations once released from catastrophes.

The absolute dating of rock art motifs in CH1 sheds light on the socioecological and adaptive context for the emergence of rock art in Patagonia during the arid mid-Holocene. Our results show an enduring first rock art style with a recurrent motif first recorded at 8.2 ka and painted over 3 ka. Paleoecological evidence from the South American deserts shows that conditions were drier and more unpredictable during this period, while human populations were either stagnant or more likely experiencing repeated crashes. In this context, we have presented a parsimonious interdisciplinary explanation where rock art tied to a site turned by transgenerational practices into a keystone cultural place facilitated social and biological connectivity in a sparsely populated high-cost landscape. Our secure chronometric results allow including South America in growing discussions about the emergence of rock art (*1*, *4*, *5*, *7*, *9*, *99*, *100*). Because rock art can fulfill various roles in diverse socioecological contexts (*96*), a multiscalar approach combining exhaustive rock art and deep-time place biographies is best equipped to address the socioecological conditions witnessing its emergence (*99*, *100*). Building social resilience to environmental change is one of the main challenges faced by humanity today. While its severity may suggest that this is unprecedented, human societies have confronted a myriad of socioecological challenges during the Holocene (*30*, *101*). We need to enhance our strategies to learn about human resilience from past experiences as contained in the archaeological record of long-term failure and success (*101*).

## MATERIALS AND METHODS

### Rock art characterization

We implemented a multiscalar and multidimensional holistic approach to the study of rock art emphasizing three complementary analytical dimensions: formal, archaeometric, and contextual (*28*). The formal dimension involved the study of the images themselves, their supports, and the relationships they present to each other through analytical units such as the motif. The characterization of the motifs contemplated morphology, technique, colors, and fading degrees. This task was undertaken after an exhaustive documentation in the field using noninvasive technologies and in the laboratory with the help of a digital enhancing program (Dstrech) (*102*). Motifs with shared characteristics were grouped in motif types to build regional typologies, and macroregional stylistic correspondences were established through the presence of images with similar morphologies in neighboring regions. The archaeometric analytical dimension included multiple physicochemical analyses such as microscopy, Raman, and SEM-EDX to generate independent results for each motif (*13*, *28*). Last, the contextual dimension encompassed the study of rock art in relation to other archaeological proxies at different spatial and temporal scales, which entail different behavioral resolution. This allowed analyzing rock art production and use in relation to its spatiotemporal context and generate subsequent interpretations about the strategies of information exchange and social interaction through visual communication (*28*).

### Archaeometric characterization

Samples were taken following established sampling protocols (*103*). Seeking to minimize damage to the images, we selected for sampling areas of the paintings where small chips were present due to natural exfoliation and that we judged free of possible contamination from soot or superimpositions. Millimetric portions of paint were removed by slight prying using a scalpel, the sterile blade of which was changed after each pigment extraction. Each sample was deposited onto a sheet of sterile aluminum foil that was then sealed on site and placed in a plastic sample bag to avoid possible alterations during transport to the laboratory. The optical microscopies were captured by a trinocular microscope, Optika brand, model SZM-2-LE, with microscopic approximation capacity, magnifying from ×0.7 to ×90. The images were processed by the Optika Vision Lite 2.1 and OptikaView7 software. Raman spectra of samples were recorded with a Raman Renishaw InVia Reflex apparatus, equipped with the 532-, 633-, and 785-nm laser lines, a Leica microscope, and an electrically cooled charge-coupled device detector. The instrument was calibrated using the 520 cm^−1^ line of a Si wafer and a 50× objective. Its resolution was set to 4 cm^−1^, and 1 to 10 scans of 10 to 50 s each were averaged. Spectra were recorded in the 200 to 1800 cm^−1^ region on several points of each sample. To count as a valid result, at least three identical spectra needed to be acquired on different analysis points. The laser power was set between 10 and 100 mW. Spectral scanning conditions were chosen to avoid sample degradation and photodecomposition; the 785-nm laser line was used. Data were collected and plotted (800 to 1800 cm^−1^) using the programs WIRE 3.4, GRAMS 9.0, and OriginLab Pro 2016. The identification of the peaks was done using the RRUFF database (available online at http://rruff.info/). After this first analysis, samples were included in resin (epoxy resin) for stratigraphic observation and elemental characterization. For the latter, images and spectra were obtained with SEM-EDX with a JEOL scanning electronic microscope, model JCM-6000, magnifying ×40 to ×850 at 15 keV.

### AMS radiocarbon dating and Bayesian analysis

The charcoal samples were analyzed at the Center for Applied Isotope Studies at the University of Georgia, USA. The samples were treated following the acid/alkali/acid protocol involving three steps: (i) an acid treatment (1 N HCl at 80°C for 1 hour) to remove secondary carbonates and acid-soluble compounds, (ii) an alkali (NaOH) treatment, and (iii) a second acid treatment (HCl) to remove atmospheric CO_2_. Samples were thoroughly rinsed with deionized water between each step, and the pretreated samples were dried at 105°C. For accelerator mass spectrometry analysis, the cleaned samples were combusted at 900°C in evacuated/sealed ampules in the presence of CuO. The resulting carbon dioxide was cryogenically purified from the other reaction products and catalytically converted to graphite using the method of (*104*). Graphite ^14^C/^13^C ratios were measured using the CAIS 0.5 MeV accelerator mass spectrometer. The sample ratios were compared to the ratio measured from the Oxalic Acid I standard (NBS SRM 4990). The sample ^13^C/^12^C ratios were measured separately using a stable isotope ratio mass spectrometer and expressed as δ^13^C with respect to Protein Data Bank (PDB), with an error of <0.1‰. The quoted uncalibrated dates have been given in radiocarbon years before 1950 (years B.P.), using the ^14^C half-life of 5568 years. The error is quoted as 1 SD and reflects both statistical and experimental errors. The dates have been corrected for isotope fractionation. The ^14^C dates were calibrated in OxCal (*60*) version 4.4 using the SHCal20 calibration curve (*105*). Bayesian phase analysis was conducted in OxCal 4.4 available at https://c14.arch.ox.ac.uk/oxcal.html (*60*, *106*). The code used is available as supplementary text S1. Each archaeological phase was initially defined on the basis of previously published chronostratigraphic and contextual evidence for CH1 (*26*, *34*, *35*, *37*, *107*, *108*). We estimated the start, end, and interval for each phase. The latter is considered the most appropriate estimator of the duration of a cultural event of interest (*60*, *109*). All modeled/calibrated estimates are noted at 95.4% confidence intervals.

### Paleoenvironmental/climatic analysis

The paleoenvironmental/climatic analysis is based on the revision of previously published records from northern Patagonia (34° to 36°S) dating back to the Late Pleistocene (*27*, *63*, *64*, *75*, *76*, *110*). Compared to a recent synthesis (*27*), we include the recently published record of Laguna El Sosneado (34.8°S) (*110*), as well as a more detailed analysis of the Mallin Vaca Lauquen (MVL) record (36.9°S) (*64*). A moisture pollen index (PML) from the MVL record is calculated here according to the following formula: PMI_MVL_ = *x*/*y*, where PMI_MVL_ is the moisture index of MVL at a given time, *x* is the percentage of Poaceae for each sample, and *y* is the percentage of *Nothofagus* spp. for each sample. The pollen percentages of *Prumnopytis andina* were plotted as a proxy of “cold forest,” while the percentages of Caryoplyllaceae are used as a seasonality proxy based on the interpretation provided in (*64*). The raw pollen data of MVL were downloaded from the Latin America Pollen Database available here: https://latinamericapollendb.com/.
