# Supplementary material for: Earliest directly dated rock art from Patagonia reveals socioecological resilience to mid-Holocene climate
Source: Sci Adv. 2024 Feb 14;10(7):eadk4415. doi: 10.1126/sciadv.adk4415 (PMC10866545; doi:10.1126/sciadv.adk4415)
Supplement: Supplementary file 1 — Figs. S1 to S7 Tables S1 and S2 Supplementary Text S1 [file sciadv.adk4415_sm.pdf]

Science Advances
AAAS

Supplementary Materials for

Earliest directly dated rock art from Patagonia reveals socioecological resilience to mid-Holocene climate

Guadalupe Romero Villanueva *et al.*

Corresponding author: Ramiro Barberena, ramidus28@gmail.com

Sci. Adv. **10**, eadk4415 (2024)
DOI: 10.1126/sciadv.adk4415

This PDF file includes:

Figs. S1 to S7
Tables S1 and S2
Supplementary Text S1

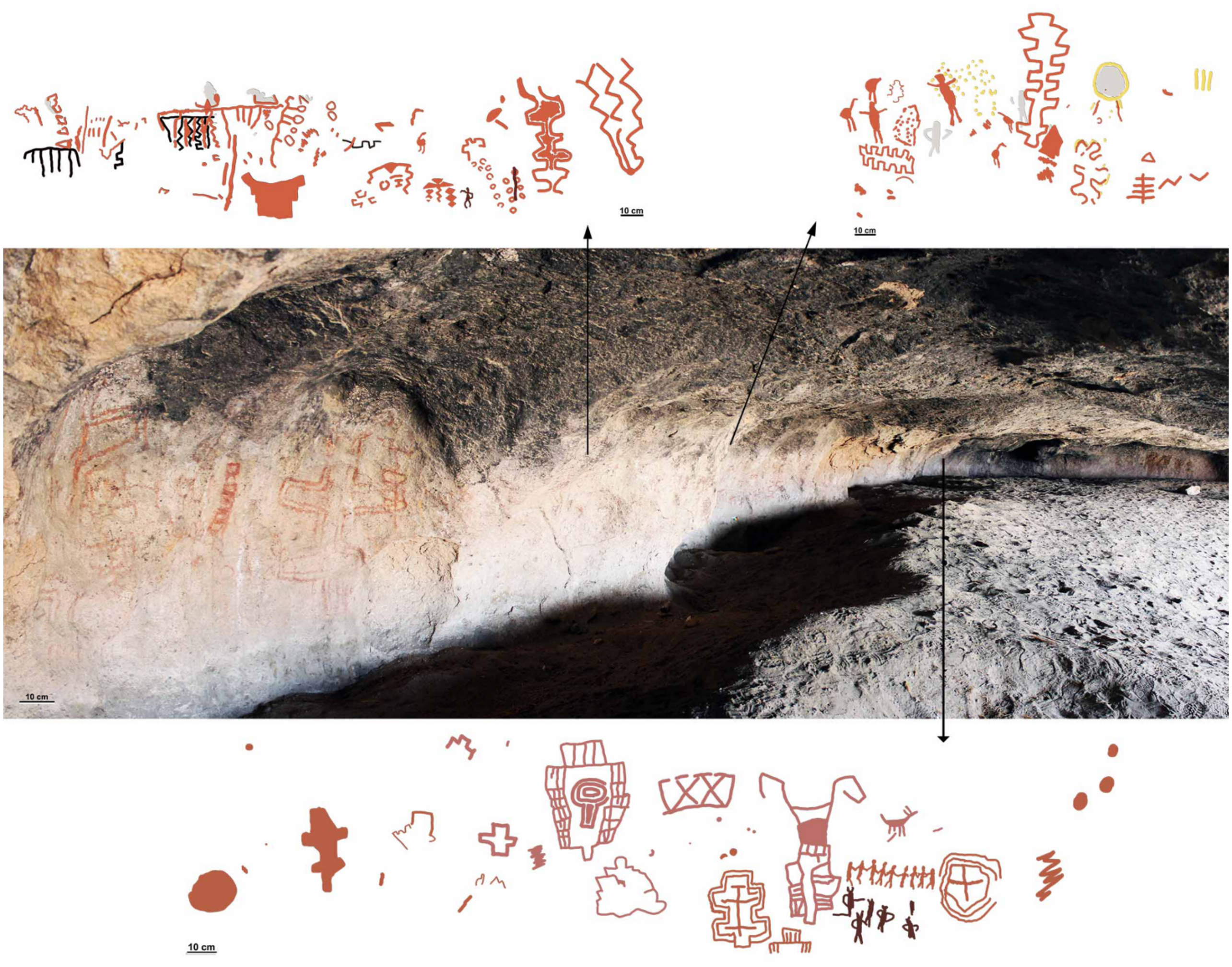

Fig. S1. Examples of the rock art of CH1. Each tracing has a 10cm scale bar. Photo and digital tracing credits: GRV.

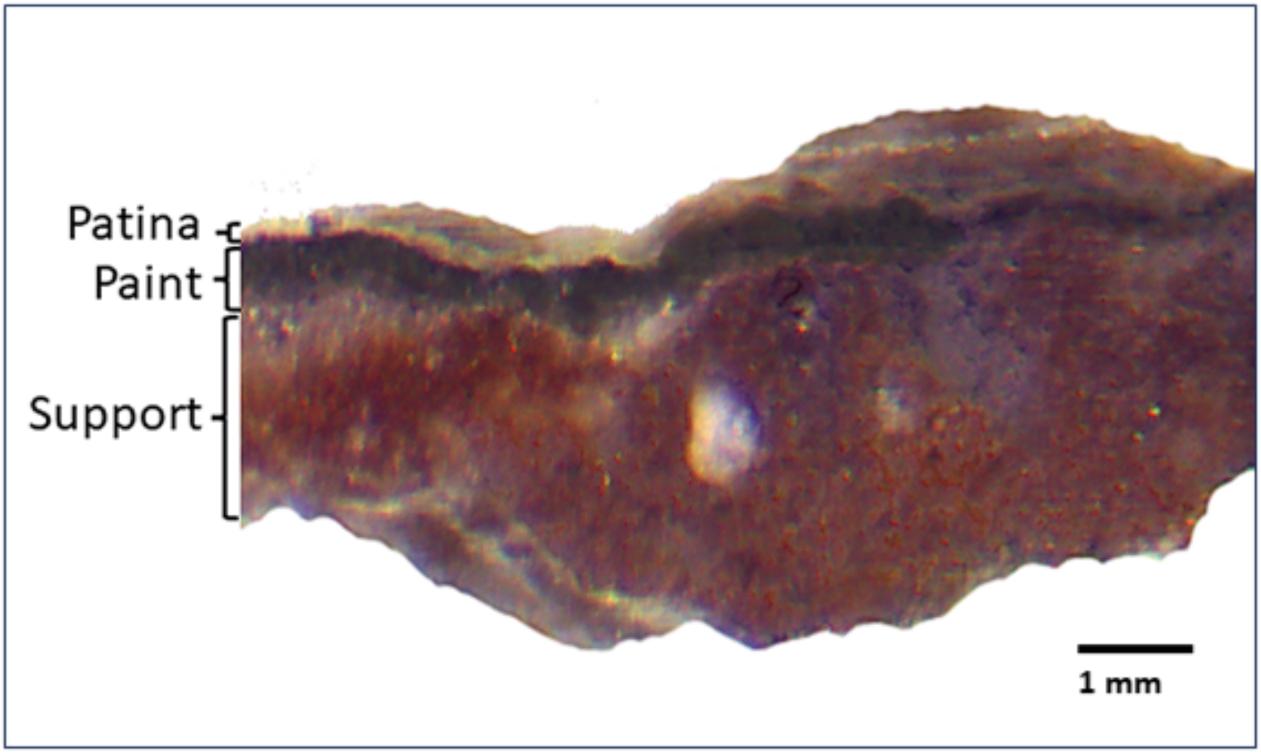

Fig. S2: Cross section microphotography of sample CH1-AMS1 embedded in resin showing three differentiated layers. From the bottom, we distinguish the bedrock support, the black pictorial layer, and a thin layer of patina or varnish. Credits: MS, GRV.

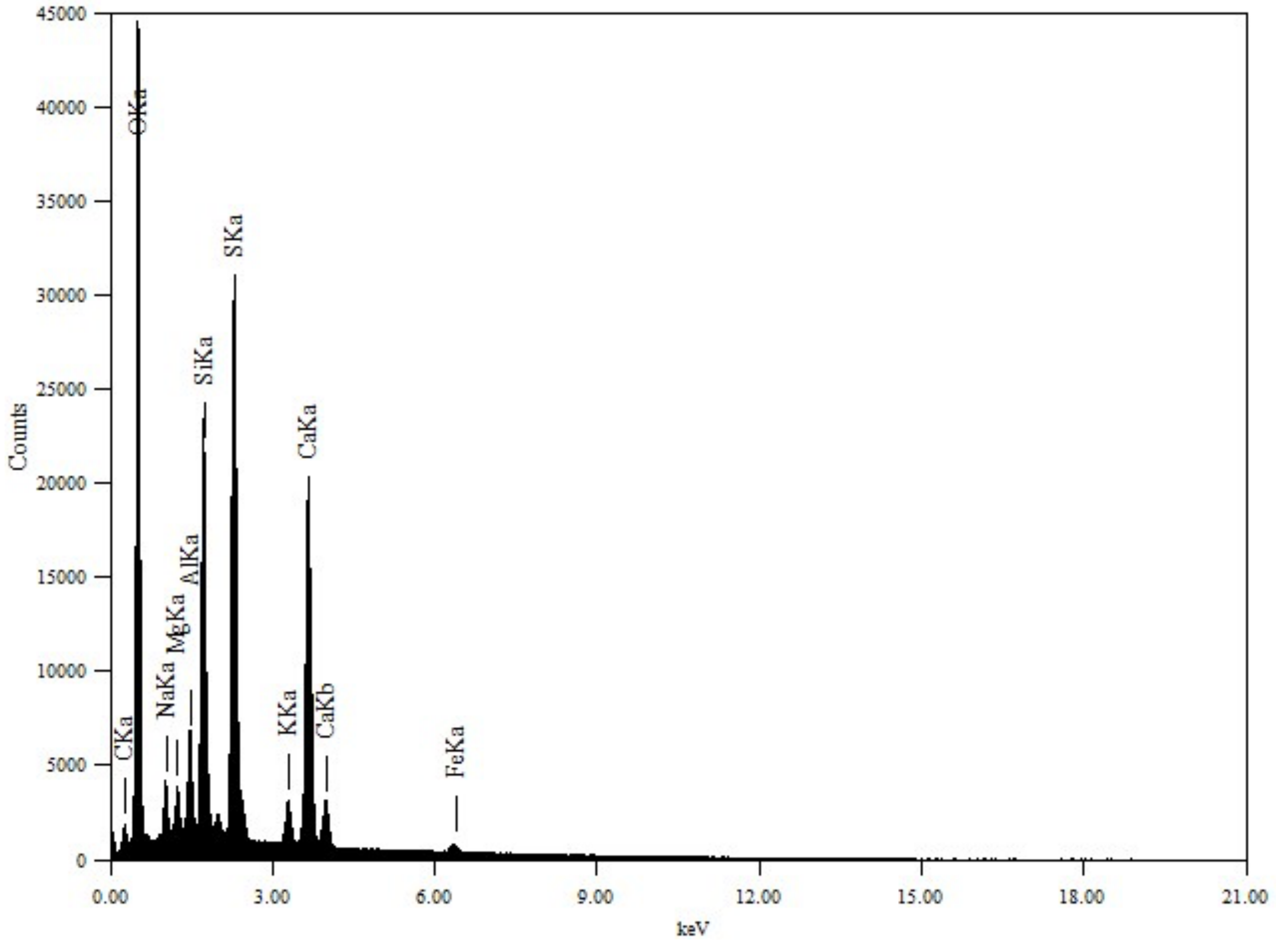

Fig. S3A. Total spectrum by SEM-EDX of sample CH1-AMS1. We can observe the presence of C, associated with Si, Al, Mg, Na, and K, and interpreted as an aluminosilicate matrix; and sulfur, and calcium –probably a salt–. Credit: MS.

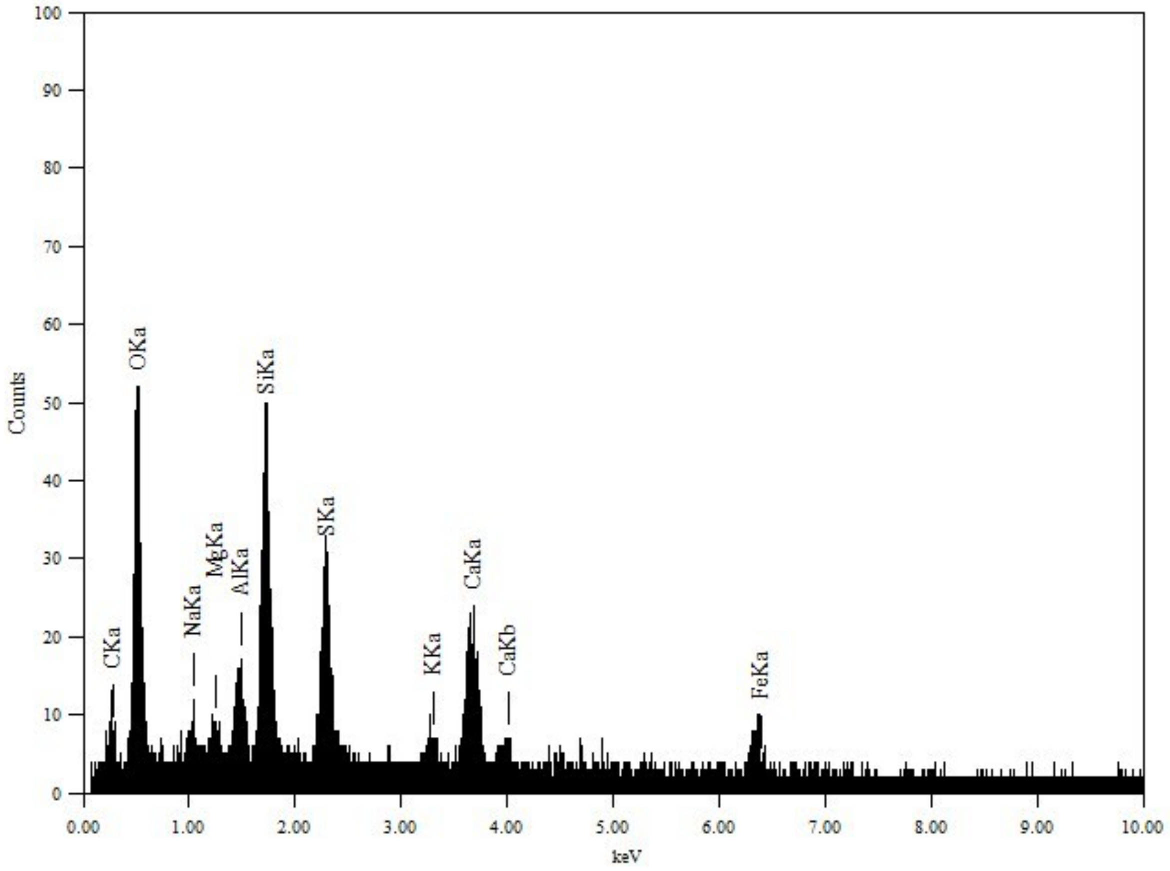

Fig. S3B. Total spectrum by SEM-EDX of sample CH1-AMS2. We observe the presence of C, associated with Si, Al, Mg, Na, and K, and interpreted as an aluminosilicate matrix; and sulfur and calcium –probably a salt–. Credit: MS.

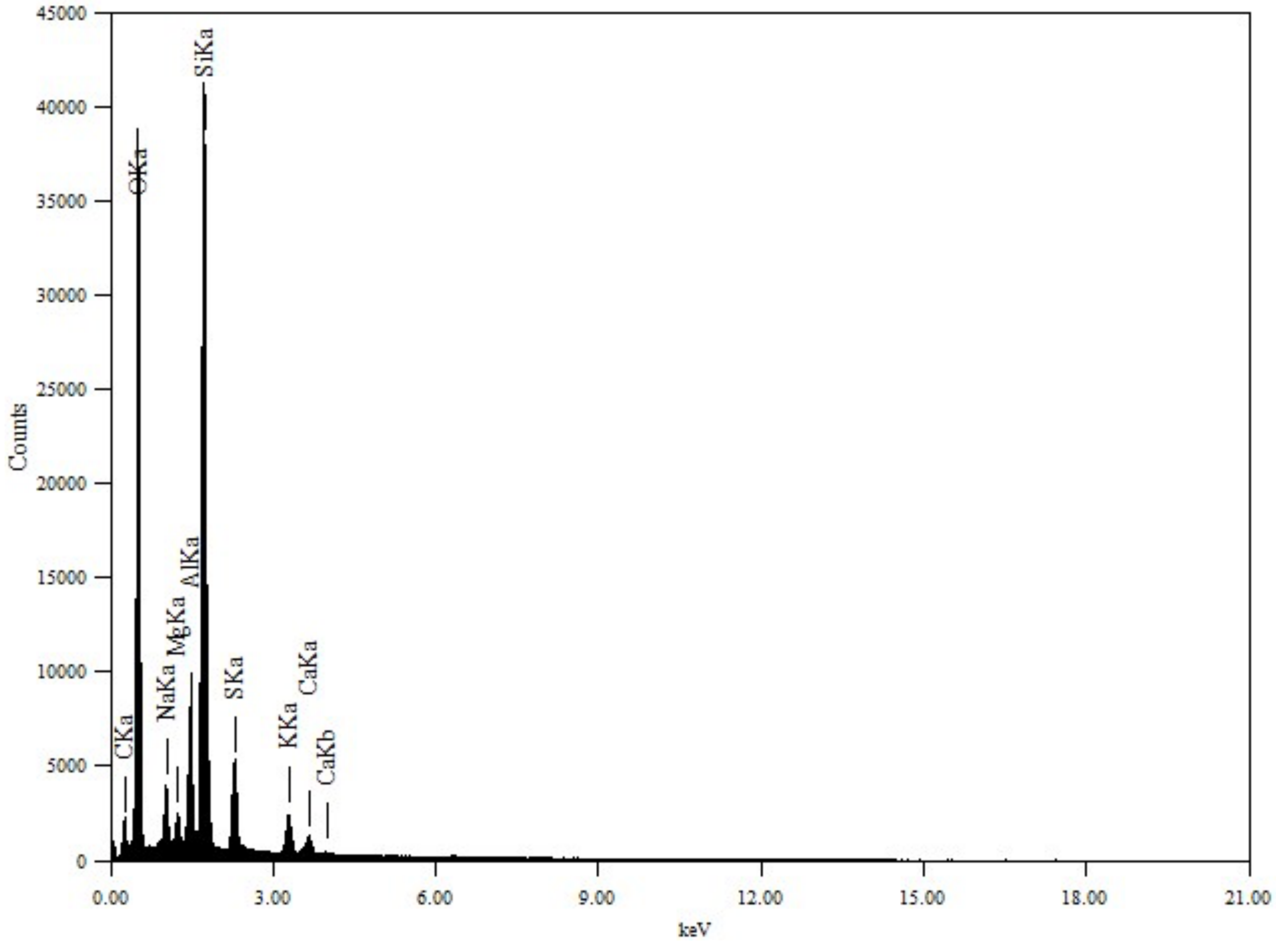

Fig. S3C. Total spectrum by SEM-EDX of sample CH1-AMS4. We can observe the presence of C, associated withthe presence of Si, Al, Mg, Na and K interpreted as an aluminosilicate matrix, sulfur, and calcium –probably a salt–. Credit: MS.

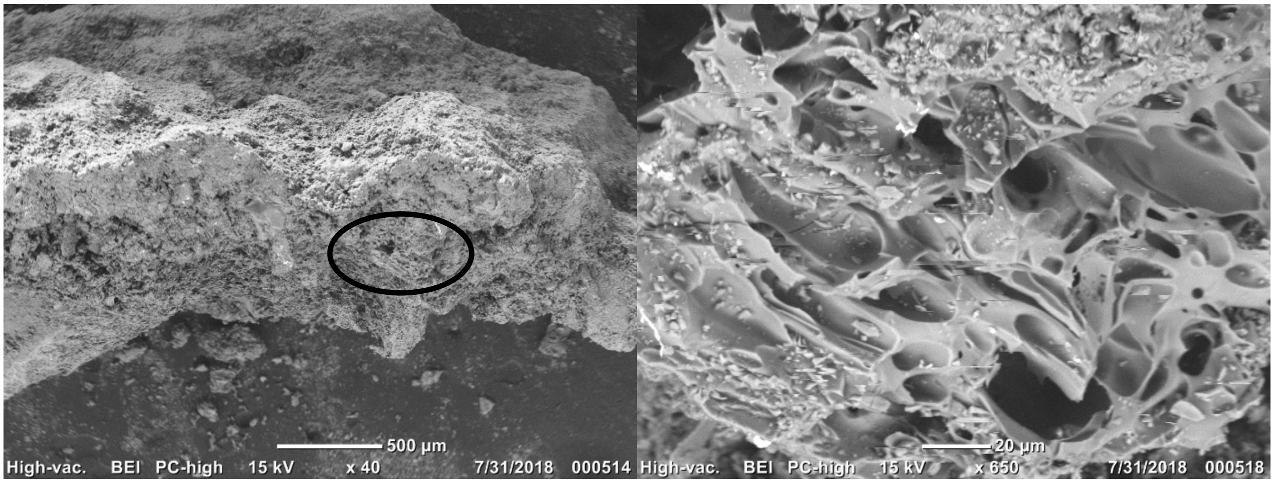

Fig. S4. SEM-EDX images of sample CH1-AMS3. The image to the left (x40) shows the presence of plant cells at the center included in other mineral concretions. The image to the right (x650) shows plant cells in more detail. Photo and digital credits: MS.

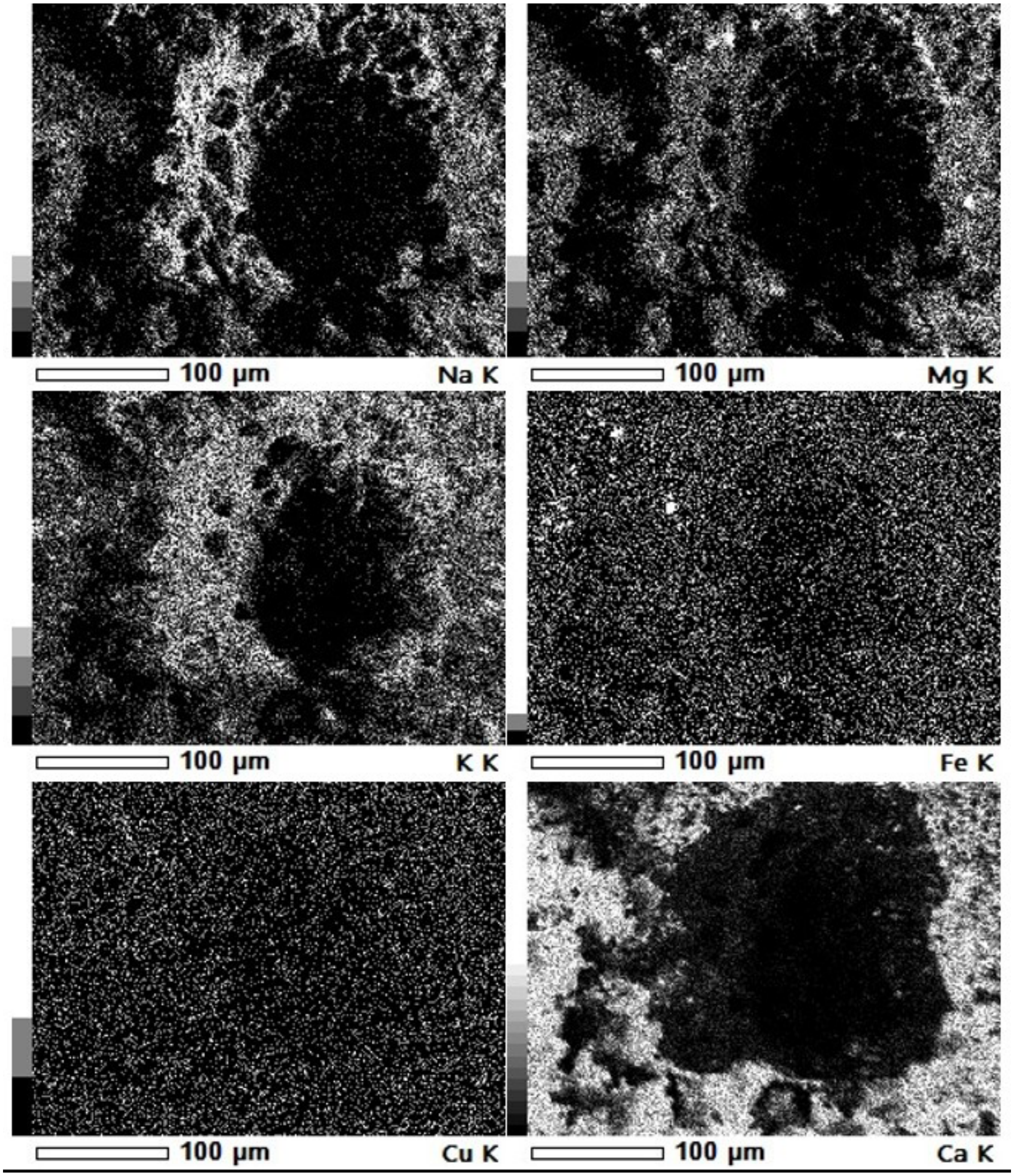

Fig. S5. Cartography with SEM-EDX of sample CH1-AMS2. In each image, lighter zones indicate the presence of the chemical element mentioned below (continued on next page). Credits: MS.

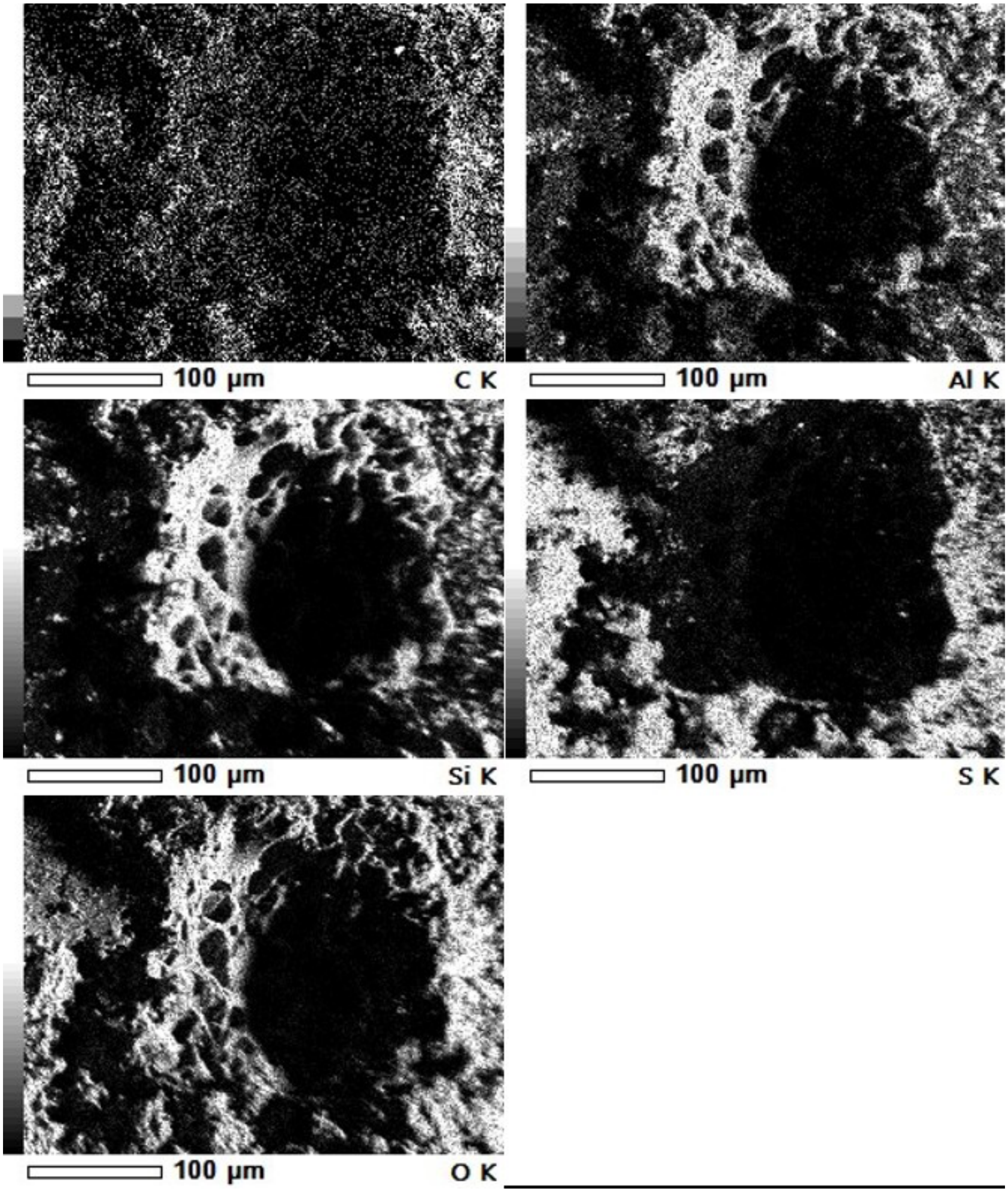
100 µm
C K
100 µm
Al K
100 µm
Si K
100 µm
S K
100 µm
O K

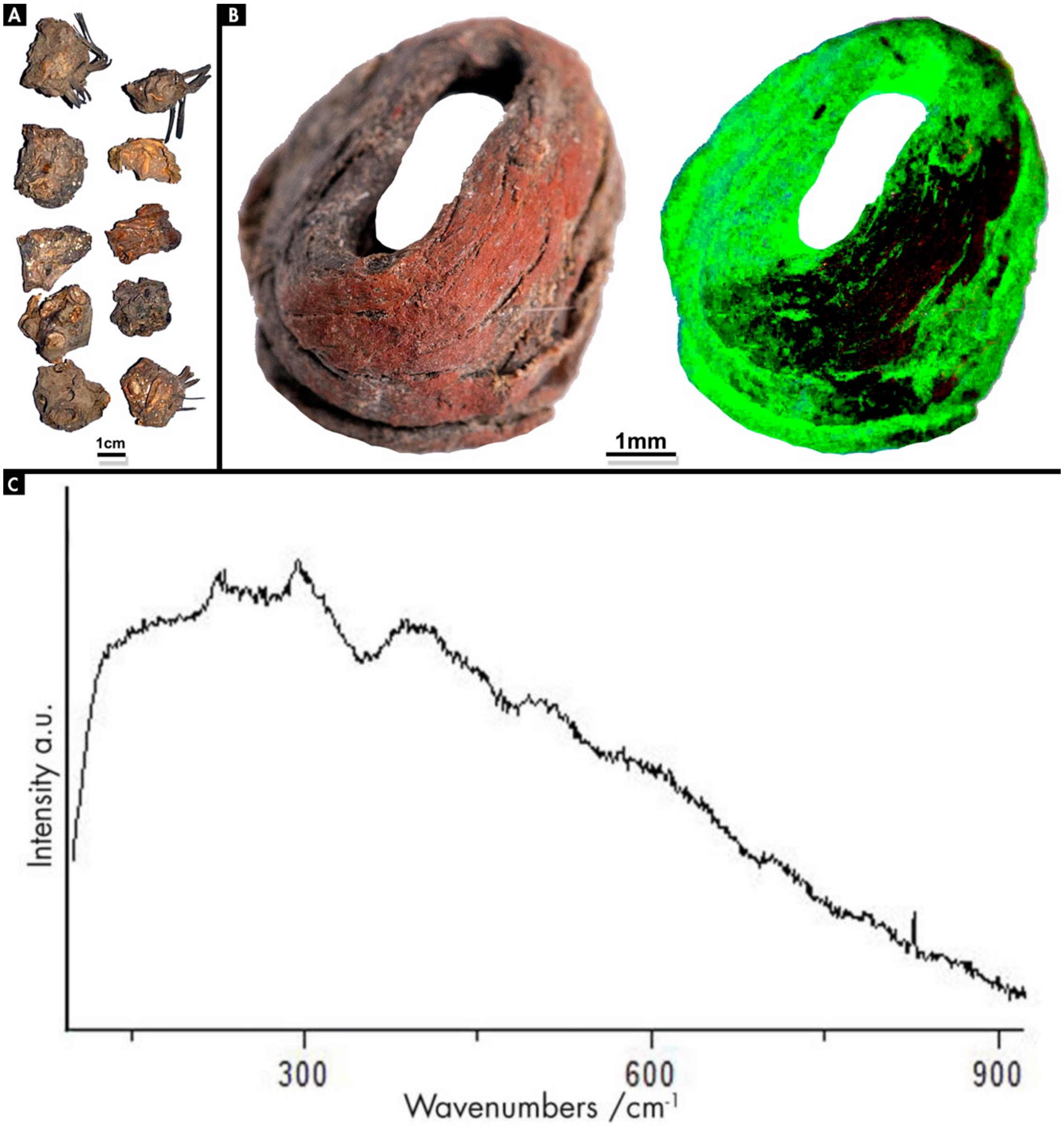

Fig. S6. ***Maihuenopsis darwinii*** **recovered at Cueva Huenul 1 site. A:** macroremains. **B:** Areola of specimen #MV2 with red coloring substance (left: original photo; right: photo digitally enhanced with D-Stretch_crgb) (*100*). **C:** RAMAN spectrum of the red pigment consistent with hematite. Credits: GRV, MS.

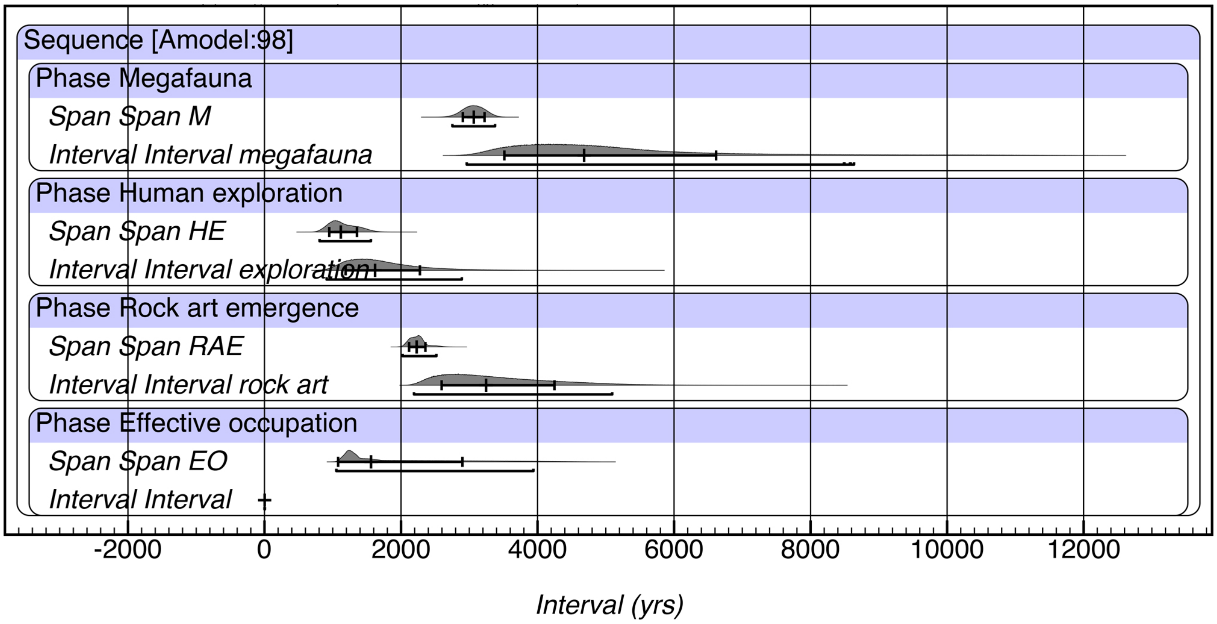

Fig. S7. Modelled intervals of the occupational phases at Cueva Huenul 1 site. Produced with OxCal v4.4.4 (*111*) with atmospheric data from (*105*).

Name	14C age	Error	Lab Code	Dated material	Unmodelled (BP)				Modelled (BP)				Indices<br>Amodel 98.2				
					sigma	median	from_95_4	to_95_4	sigma	median	from_95_4	to_95_4	Acomb	A	L	P	C
**Sequence**																	
**Boundary Start 1**									1418	17407	21343	16447					96
**Phase Megafauna**																	
**R_Date 5**	13844	75	AA85722	megafauna dung	137	16761	17015	16495	140	16734	17004	16466		98.2			99.7
**R_Date 3**	11841	56	AA85720	megafauna dung	76	13668	13789	13512	76	13674	13790	13513		100.3			99.7
**Span Span M**									159	3066	2753	3378					99.7
**Interval Interval megafauna**									1551	4683	2963	8637					99
**Boundary End 1**									566	12934	13730	11775					99.5
**Boundary Start 2**									349	11721	12501	11270					99.1
**Phase Human exploration**																	
**R_Date 10**	10155	98	AA99106	bone (*Lama guanicoe*)	216	11689	12056	11265	185	11470	11876	11246		89.8			99.5
**R_Date 1**	9531	39	AA85718	charcoal	143	10761	11071	10584	143	10761	11071	10584		99.7			99.6
**R_Date 15**	9402	60	AA102574	seed (*Neltuma* sp.)	105	10582	10760	10307	103	10583	10762	10376		100.6			99.6
**R_Date 8**	9375	91	AA99104	bone (*Lama guanicoe*)	154	10542	11056	10248	151	10549	11056	10253		101.6			99.5
**R_Date 9**	9295	90	AA99105	bone (*Lama guanicoe*)	121	10435	10663	10238	118	10454	10669	10244		100.1			99.6
**R_Date 11**	9261	66	AA99107	plant fragment (*Retanilla* sp.)	92	10392	10566	10243	91	10412	10570	10248		98.7			99.7
**Span Span HE**									203	1118	808	1559					99.6
**Interval Interval exploration**									544	1620	910	2891					99.1
**Boundary End 2**									356	10162	10515	9263					98.6
**Boundary Start 3**									619	8171	9706	7582					99
**Phase Rock art emergence**																	
**R_Date 21**	6830	50	UGAMS 2970	amrophous carbon	46	7636	7737	7519	45	7633	7734	7518		100.3			99.8
**R_Date 20**	5360	50	UGAMS 2969	amrophous carbon	83	6105	6276	5942	83	6106	6276	5942		99.8			99.8
**R_Date 16**	4786	46	AA102575	pigmented plant fragment (*Senna aphylla*)	79	5493	5588	5325	76	5501	5589	5326		101.4			99.9
**R_Date 18**	4730	110	UGAMS 2967	amrophous carbon	150	5412	5652	5047	132	5443	5657	5058		105.1			99.5
**Span Span RAE**									120	2230	2024	2518					99.8
**Interval Interval rock art**									826	3246	2191	5094					99.3
**Boundary End 3**									499	5074	5553	3849					99.6
**Boundary Start 4**									917	4216	5399	2058					98.9
**Phase Effective occupation**																	
**R_Date 13**	1753	47	AA99110	bone (*Lama guanicoe*)	57	1623	1729	1526	57	1623	1729	1526		99.8			99.8
**R_Date 7**	1590	46	AA99103	bone (*Lama guanicoe*)	55	1440	1535	1318	55	1439	1536	1319		99.9			99.8
**R_Date 17**	1462	33	D-AMS 018771	decorated bone (*Lama guanicoe*)	27	1320	1370	1280	26	1320	1370	1280		99.2			99.8
**R_Date 4**	1416	37	AA85721	grasses	45	1288	1353	1178	45	1288	1353	1178		99.3			99.9
**R_Date 12**	1269	46	AA99109	bone (*Lama guanicoe*)	67	1136	1271	993	67	1137	1271	993		99.5			99.9
**R_Date 22**	983	29	D-AMS 033195	*Zea mays*	41	853	924	772	41	853	924	773		99.8			99.9
**R_Date 14**	541	42	AA102573	*Lagenaria* sp.	27	525	625	492	27	525	625	491		99.8			99.8
**R_Date 6**	373	43	AA99102	bone (*Lama guanicoe*)	53	394	491	311	53	394	491	311		99.8			99.9
**Span Span EO**									910	1561	1053	3940					99.7
**Interval Interval**									0	3	0	5					100
**Boundary End 4**									1280	1875	4323	-12					99.1

Table S1. Radiocarbon dates for Cueva Huenul 1 site and OxCal (60) Bayesian modeling for phase start, end, and intervals.

ID (Fig. 1)	Site	Latitude (°S) Longitude (°W)	Type of archive	Source
1	Laguna El Sosneado	34.84/69.91	Pollen and charcoal	(*110*)
2	Río Valenzuela/Grande	32.35-32.29/ 70.51-70.52	Geomorphological	(*75*, *76*)
3	Huenul	36.96/68.82	Pollen and plant macro-remains	(*27*)
4	Laguna del Maule	36.05/70.49	Multiproxy (pollen for this paper)	(*63*)
5	Mallín Vaca Lauquen	36.85/71.04	Pollen and charcoal	(*64*)

Table S2. Information of those paleoecological/paleoclimatical sites included in the macro-regional discussion. Sources cited in the main text.

Suppldementary text S1. OxCal (*111*) code for the phase modelling of Cueva Huenul 1 site.

```
Options()
 {
  Curve="shcal20.14c";
  BCAD=FALSE;
SD2=TRUE; };
 Plot()
 {
  Sequence()
  {
   Boundary("Start 1");
   Phase("Megafauna")
   {
    R_Date("5",13844,75);
    R_Date("3",11841,56);
    Span("Span M");
    Interval("Interval megafauna");
   };
   Boundary("End 1");
   Boundary("Start 2");
   Phase("Human exploration")
   {
    R_Date("10",10155,98);
    R_Date("1",9531,39);
    R_Date("15",9402,60);
    R_Date("8",9375,91);
    R_Date("9",9295,90);
    R_Date("11",9261,66);
    Span("Span HE");
    Interval("Interval exploration");
   };
   Boundary("End 2");
   Boundary("Start 3");
   Phase("Rock art emergence")
   {
    R_Date("21",6830,50);
    R_Date("20",5360,50);
    R_Date("16",4786,46);
    R_Date("18",4730,110);
    Span("Span RAE");
    Interval("Interval rock art");
   };
   Boundary("End 3");
   Boundary("Start 4");
   Phase("Effective occupation")
   {
    R_Date("13",1753,47);
```

```
   R_Date("7",1590,46);
   R_Date("17",1462,33);
   R_Date("4",1416,37);
   R_Date("12",1269,46);
   R_Date("22",983,29);
   R_Date("14",541,42);
   R_Date("6",373,43);
   Span("Span EO");
      Interval("Interval");
   Boundary("End 4");
  };
 };
};
```

Provenance

The samples analyzed in this research were collected by GRV (CONICET) and RB (Universidad Católica de Temuco, CONICET) during authorized archaeological field research conducted in 2017. All the details on the identification, validation, and radiocarbon dating of these samples are presented in the methods section of this manuscript, as well as in this Supplementary Materials.
